# Exploring the Meaning of Cultural Competence Among Undergraduate Nursing Students

**DOI:** 10.1155/nrp/3963409

**Published:** 2026-02-15

**Authors:** Wai Shan Shirley Huang, Daniel Terry, Blake Peck

**Affiliations:** ^1^ Institute of Health and Wellbeing, Federation University Australia, Ballarat, Victoria, Australia, federation.edu.au; ^2^ School of Nursing and Midwifery, University of Southern Queensland, Toowoomba, Queensland, Australia, usq.edu.au; ^3^ Centre for Health Research, University of Southern Queensland, Toowoomba, Queensland, Australia, usq.edu.au

**Keywords:** communication, cultural competence, education, intercultural, mixed-methods design, nursing, student

## Abstract

**Introduction:**

The cultural backgrounds of Australian populations are increasingly diverse due to trends in globalisation and migration. Specifically, within healthcare environments, the rapid growth of multicultural individuals necessitates a change in nursing practice. Healthcare professionals require culturally competent knowledge, skills, and attitudes to accommodate individualised healthcare needs of a diverse population. Therefore, preparing student nurses with an adequate level of cultural competence remains essential in nursing education.

**Background:**

Previous studies indicated that the meaning of cultural competence is ambiguous. Additionally, there is a paucity of research examining the definitions of cultural competence among students with heterogeneous cultural backgrounds. Thus, this study aims to explore undergraduate nursing students’ perceptions and learning experiences of cultural competence, with attention to the personal and educational factors that shape their understanding.

**Materials and Methods:**

An explanatory sequential mixed‐methods research design was used, and data collection included a survey followed by a series of interviews with undergraduate students. A total of 25 nursing students had completed a full set of Cultural Competence Assessment Tool questionnaires, with five participating in a one‐on‐one interview. Both descriptive and inferential statistical methods were used to analyse the survey data, while the interviews were analysed using a thematic analysis.

**Results:**

Overall, students have a moderate level of cultural competence and scored low on cultural sensitivity. Additionally, three recurring themes were identified in the qualitative results and encompassed understanding cultural competence is a lifelong journey, factors influencing students’ interpretations of cultural competence, and learning cultural competence.

**Conclusion:**

The findings highlight intercultural communication barriers and social segregation as contributing factors to demographic differences in cultural competence. The insights gained may benefit cultural content development in higher education curricula, specifically in nursing contexts. Future research may explore the benefits of alternative pedagogical strategies in enhancing students’ cultural competency.

## 1. Introduction

Cultural competency is regarded as an essential component in the curriculum of tertiary nursing education [1]. In Australia, there is a rich mix of cultural backgrounds and heritage, with the number of international migrants continuing to increase in recent decades. Approximately 30% of Australians were born overseas, and more than 50% are second‐generation migrants [2]. A previous study found that patients from culturally and linguistically diverse backgrounds encounter a range of challenges when engaging with healthcare services, including communication barriers and cultural misunderstandings, which may contribute to health disparities [3]. Therefore, cultural competence, a core value in the nursing profession, is expected by the public, and ensuring culturally safe and respectful practice during clinical care is a fundamental component of nursing professional ethics and responsibilities [4]. A mixed‐methods study by Wu et al. [5] found that more than 90% of medical and dental students perceived that it is important for healthcare providers to be culturally competent. As such, the nursing education system, likewise, plays a pivotal role in improving student nurses’ cultural competence [6]. Moreover, adequate cultural knowledge and competence in cultural skills are essential for nursing students during clinical practice [7].

Cultural competency among healthcare professionals is associated with increased patient satisfaction, the promotion of human rights, and reduced health inequalities from a global perspective [8]. Further, with adequate cultural competence training, healthcare providers are better able to apply a patient‐centered approach when conducting a behavioural analysis and developing a treatment plan [9]. More specifically, when adequate cultural competence training is provided, nurses become more skilled in intercultural communication during patient interactions such as medical history taking, which results in improved patient satisfaction and treatment adherence [9].

According to the Australian Department of Education Skills and Employment [10], approximately 32% of the higher education student enrolments are international students, and many will become an integral part of the national nursing workforce in the future. Given that the international students often have cultural and language backgrounds that differ from those of domestic students, the distinctive cultural behaviours and linguistic backgrounds may create barriers to developing cultural competency in clinical practice and academic learning experiences [11]. Unfamiliarity with cultural awareness has been shown to lead to intercultural communication challenges for both local and international students, leading to poor cultural knowledge, cultural insensitivity, frustration and uncertainty during reflective practice, and patient mistrust [12]. Therefore, it is crucial to assist nursing students in developing cultural competence, as this enhances their capacity to interact more effectively with patients and colleagues at the forefront of clinical settings [13].

Although many current nursing programmes have incorporated transcultural nursing education into their curricula, there is a palpable gap in both the content and delivery of cultural competence [14]. Students have indicated that the cultural content in their nursing curricula remains inadequate and inconsistent with real practice [14]. As a result, students feel inadequately prepared and lack confidence in meeting the diverse needs of patients, which hinders their ability to deliver culturally and linguistically appropriate care [14, 15].

Despite the growing emphasis on cultural competence in nursing education, there remains a limited understanding of how students from diverse cultural backgrounds perceive and learn cultural competence within the same educational context. Most existing studies focus on measuring competence levels or evaluating specific interventions, with less attention to students’ lived experiences and meaning‐making processes of students navigating multicultural academic and clinical environments. Within this context, the aim of this study was to explore undergraduate nursing students’ perceptions and learning experiences of cultural competence, with attention to the personal and educational factors that shape their understanding. The research questions encompassed (1) How do domestic and international undergraduate nursing students define cultural competence? (2) What are the factors that impact students’ interpretations of cultural competence? As such, the study contributes new knowledge to the field by capturing the nuanced, student‐driven perspectives that are often underrepresented in the literature.

## 2. Materials and Methods

The study used an explanatory sequential mixed‐methods research design to explore how domestic and international undergraduate nursing students conceptualise and define cultural competence and the factors that influence their interpretations of it. The Cultural Competence Assessment Tool (CCATool)–Student Nurse Specific questionnaire [16] was used to assess students’ level of cultural competence and to investigate the factors that may influence their definitions of cultural competence. In addition, individual semistructured interviews were employed, as they yield further and richer data to answer the research questions. The sequential design allowed the questionnaire results to inform the interviews. Specifically, lower scores in cultural sensitivity and variations across demographics guided the refinement of interview questions to explore students’ experiences and perceptions in greater depth. This approach sought to expand on personal experiences and understandings of cultural competence, as well as on students’ transcultural experiences in their academic learning and clinical practice [17].

### 2.1. Sample and Setting

All third‐year undergraduate nursing students in 2022 (*n* = 683) were invited to participate, and students self‐selected to participate in the study. Only third‐year students were included because they have greater exposure to culturally diverse populations as part of their 800 h of Professional Experience Placement, a compulsory element of clinical practice subjects required for programme completion. The participants were recruited through an advertisement posted on the university’s learning management system, which included a plain language information sheet and a link to access the online questionnaire. Reminders regarding the study were sent out each week for 4 weeks, starting with the initial advertisement posted on the learning management system.

### 2.2. Data Collection

#### 2.2.1. Quantitative Data Collection

Participants who agreed to participate were invited to complete a self‐reported online questionnaire using Qualtrics. The data were collected using the CCATool–Student Nurse Specific, originally developed by Papadopoulos [16]. The CCATool wording was slightly modified for an Australian audience and context, with the tool developers’ approval. The questionnaire consists of two sections; the first section contains four crucial elements that help to address the research questions: cultural awareness, cultural knowledge, cultural sensitivity, and cultural practice [18]. Cultural awareness refers to the level of self‐awareness and exploration of our own cultural background and cultural identity. This process helps us understand our biases, prejudices, and assumptions and be aware of our own cultural heritage [19]. Cultural knowledge refers to the process of acquiring and deepening understanding of diverse cultural and ethnic groups [20]. Cultural sensitivity is the attempt to understand and appreciate cultural differences [21]. Cultural practice involves respecting individuals’ beliefs, traditions and customs associated with their cultural group to support the equitable delivery of care. Each section contains 10 statements on a 4‐point scale (1 = completely disagree, 2 = disagree, 4 = agree, 5 = completely agree) [16, 18]. It must be noted that, within Papadopoulos’ [16] CCATool, there is no neutral option (3) on the scale, and students must either disagree or agree with the statements. For the scoring method, each statement is assigned a score based on the response rating. Codes 4 or 5 receive 1 point, while codes 2 or 1 receive 0 points. Thus, the total possible score is 40 points. The following scoring instructions outline the method to determine levels of cultural competence based on respondents’ ratings across four sections [16]:

1. Cultural incompetence: score less than 5 in cultural awareness, irrespective of the score in other sessions
2. Cultural awareness: score of 5 or more in cultural awareness, without necessarily having all the statements correct in the other sections
3. Cultural safety: a score of 5 or more in cultural awareness and all the generic statements are correct in the other sections
4. Cultural competence: a score of 10 in all sections

The second section included questions to elicit information on the students’ demographic characteristics, the employment status of the participants, and their clinical experiences across the Bachelor of Nursing programme.

The instrument was based on the original tool developed and validated by Papadopoulos [16]. Minor wording changes were made to align with the language and expressions used in the Australian context, with the author’s approval ([16], personal communication). It has good internal consistency with Cronbach’s alpha, for which the scales of cultural awareness, cultural knowledge, cultural sensitivity, and cultural practice were 0.786, 0.734, 0.643, and 0.826, respectively [22]. The questionnaire took about 20–30 min to complete. To ensure the study had adequate statistical power to detect a 5% absolute difference within and between groups, the calculated sample size of *n* = 67 was calculated. This calculation was based on an alpha level of 0.10 and a margin of error of ±10%, which were selected to balance statistical sensitivity with feasibility, given the expected response rate. The 5% threshold was chosen to reflect a meaningful difference in attitudes or experiences across groups, based on prior literature and practical significance in nursing education research [23].

#### 2.2.2. Qualitative Data Collection

At the end of the questionnaire, participants were invited to a one‐on‐one interview. Those who agreed to participate in a semistructured interview were then contacted to arrange a suitable time and date for a video conference interview. Within qualitative studies, there is ongoing debate concerning a minimum sample size, with some suggesting at least 12 participants [24], while others have indicated between five and 25 participants may be suitable [25]. In this study, all participants who were available and willing to contribute were included, ensuring the richness and diversity of perspectives were captured as fully as possible.

While traditional qualitative approaches often rely on the concept of data saturation, where no new themes or codes emerge, Braun and Clarke [26] critique this notion within reflexive thematic analysis. They argue that saturation reflects a neo‐positivist stance and is not entirely consistent with the interpretative and iterative nature of reflexive thematic analysis. Instead, they emphasise the importance of depth, richness, and meaning‐making in the data, encouraging researchers to focus on the quality of interpretation rather than reaching a point of thematic exhaustion. Accordingly, this study did not achieve saturation in a strict sense but rather ensured data collected were sufficiently rich and nuanced to support meaningful thematic development.

The interview schedule was designed based on a thorough review of existing literature on the life and learning experiences of nursing students in relation to culture and cultural competence. Informal input from researchers was also obtained, and the questions were cross‐checked and verified with health workforce research. To maintain consistency and credibility, an interview schedule was adhered to (Supporting file 1). Each interview consisted of nine open‐ended questions and three prompting questions. Interviews began with an open‐ended question to initiate a conversation: “I am interested in your thoughts and personal experience related to cultural competency, and I want to start with a discussion about what is the first thing that comes to your mind when talking about culture?” This initial question facilitated dialogue, enabling the researcher to continue with the conversation naturally. Prompting questions until all participants had exhausted all avenues of information related to the study’s aims. All interviews were conducted by the lead researcher (SH) and lasted between 30 and 45 min. During the interview, verbal communication was digitally recorded and transcribed.

Lastly, a reflexivity journal was maintained throughout the research process. The lead researcher (SH), who has a nursing education background and experience working with culturally diverse populations, reflected on her positionality and potential influence on data collection and interpretation. To mitigate bias, an audit trail was kept, and regular peer debriefings were conducted with co‐researchers.

#### 2.2.3. Data Analysis

Quantitative data were cleaned to ensure accuracy and consistency. This involved checking for missing values, correcting errors and removing any irrelevant or duplicate entries before analysis. The data were then cross‐checked, followed by statistical analysis using IBM SPSS Statistics (Version 21). Descriptive statistics were generated from the data and used to describe the sum variables for cultural competence and each subsection. Moreover, inferential statistics, such as the Mann–Whitney *U* test, were used to compare the background variables associated with cultural competence. In addition, chi‐square (*χ*2) tests were used to determine if factors differed between groups. The level of significance was determined by a two‐tailed test *p* ≤ 0.05. Qualitative data were transcribed and analysed, and initial coding was conducted by the lead researcher (SH) using thematic analysis as described by Braun and Clarke [27]. Repeated readings of the transcripts were undertaken to identify patterns and generate inductive codes. These preliminary codes were then grouped into broader categories and potential themes. To enhance the reliability and credibility of the analysis, two additional researchers (DT and BP) independently reviewed the coded data and emerging themes. The team met regularly to discuss interpretations and resolve discrepancies, ensuring consensus on theme development. Lastly, the report was produced after conducting a final analysis of selected extracts and relating back to the research questions. Data were labelled according to the order in which the participants were interviewed (e.g., Participant 1, Participant 2, and so on).

#### 2.2.4. Ethical Considerations

The study was approved by the Human Research Committee of Federation University Australia (Reference Number: A22‐024). The research followed the ethical principles for medical research involving human subjects as outlined in the Declaration of Helsinki, and all procedures were conducted in compliance with the relevant guidelines and regulations. Implied consent was obtained from all participants who voluntarily agreed to participate in the online questionnaire, and written consent was obtained from those who agreed to be interviewed.

## 3. Results

A total of *n* = 25 students agreed to participate in the study, of which 80% (*n* = 20) were female. The mean age of participants was 21 years (SD = 10.5 years). Participants were from culturally diverse backgrounds, with 60% (*n* = 15) being of Caucasian (Australian) background. Among the remaining participants, five were from India (*n* = 5), three were from the Netherlands (*n* = 3), while the remaining were from Türkiye (*n* = 1) and Columbia (*n* = 1). Regarding the location of clinical placements, nearly half (*n* = 15, 60%) of the students were allocated to metropolitan hospitals in Victoria to undertake their Professional Experience Placement.

### 3.1. Cultural Competence

All 25 students completed the questionnaire. The sum variables for cultural competence and each subsection of cultural competence were calculated as shown in Table 1. The overall median score was 8.0 out of 10.0, indicating that students have a moderate level of cultural competence. Specifically, students demonstrated a high level of cultural awareness and cultural knowledge, a moderate level of cultural practice, but a low degree of cultural sensitivity. Additionally, the moderate variations between the mean scores of the subsections suggest that although students may achieve a high overall median score, lower scores in cultural sensitivity could negatively impact their overall level of cultural competence.

**TABLE 1:** Description of cultural competence as sum variables.

	Cultural competence	Cultural awareness	Cultural knowledge	Cultural sensitivity	Cultural practice
Median	8.0	10.0	9.0	6.0	10.0
SD	0.9	0.8	1.2	1.4	0.8
Minimum	5.0	8.0	5.0	5.0	8.0
Maximum	9.3	10.0	10.0	10.0	10.0

The variations between demographics and the level of cultural competence were examined. When examining total competence scores, there was a significant difference between males (Median = 8.50) and females (Median 7.75), *p* = 0.047, with a small effect size (0.630), indicating a modest practical difference. In addition, a significant difference in cultural sensitivity was demonstrated when comparing Australian students (Median = 8.25) and non‐Australian students (Median = 8.00), *p* = 0.003 (Table 2), which was also accompanied by a small‐to‐moderate effect size (0.257). This suggests that while the differences are statistically significant, their practical impact may be limited. It must be noted that no other significant differences were detected when comparing age groups, *p* = 0.640, as outlined in Table 2. Lastly, when examining the subscales of cultural awareness, cultural knowledge, cultural practice, and cultural competence, no significant differences were observed across demographic groups.

**TABLE 2:** Differences between cultural competence and demographic variables.

	Cultural competence					
	N	Md	Sd	Mann–Whitney *U* Test	Effect size (r)	*p* value
Gender Male Female	5 20	8.50 7.75	0.411 1.075	18.50	0.630	0.047
Age 35 years and under Over 35 years	9 16	7.75 8.25	1.317 0.827	59.50	0.174	0.640
Cultural background Australian Non‐Australian	16 9	8.25 8.00	1.128 0.718	53.50	0.257	0.003

Among those who agreed to be interviewed (*n* = 5), four were female, and two were of Indian cultural background. When exploring how undergraduate nursing students interpreted and defined cultural competence, three recurring themes were identified and encompassed the (1) understanding cultural competence is a lifelong journey, (2) factors influencing students’ interpretations of cultural competence and (3) learning cultural competence, which are discussed in detail.

### 3.2. Understanding Cultural Competence Is a Lifelong Journey

This theme outlines the students’ perceptions of cultural competence, particularly its nature as a continuous process of personal and professional growth. Participants perceived that cultural competence is an ongoing process of growth and learning, which they believed was important for being compassionate and delivering culturally competent care. Participants commented that, despite feeling culturally competent, they still recognised the importance of ongoing learning to adapt to diverse cultural contexts. They also acknowledged that continuous development in delivering culturally sensitive care is crucial in the nursing profession. Similarly, other participants shared a similar view regarding the committed dedication required to strive for cultural competence. For instance, Participant 4’s response reflected, “I view cultural competence as an ongoing journey. You know, you can never achieve a growth mindset. You can only ever strive for it. I feel like cultural competence might be a bit like that.”

Additionally, participants highlighted that intercultural learning is not simply about passively receiving information, but rather it is cultivated through meaningful interpersonal interactions. They described culture as something learnt naturally through one’s lived experiences and social environment as one grows up. Other participants similarly emphasised that personal experience and intercultural exposure are essential components in fostering cultural competence. They suggested that the attitude of being receptive to learning and aware of one’s own knowledge gaps is part of the cultural learning journey. Participants also expressed an appreciation of intercultural experiences, which enabled them to gain new perspectives and insights. By incorporating new perspectives, students could enhance their empathy and cultural awareness, ultimately improving their cultural competence. For example, Participant 4’s comment showed that learning cultural competence is a continuous process of personal and professional growth, which requires self‐awareness and reflection:

*The more experience I have working with other cultures and colleagues, the more I learn about cultural competence.* (P4)

Participants indicated that they had a good understanding of culture and cultural competence. From their perspective, they identified identity, countries of origin, family traditions and beliefs, religion, traditional customs, festivals, food, etc., as the key elements of culture. It is interesting to note that they were not able to provide concise descriptions and interpretations of cultural competence. It also emerged that participants acknowledged their limited understanding of its meaning. During the interviews, participants attempted to define cultural competence by referencing cultural elements. Notably, only one participant was able to describe cultural competence as involving understanding and respect for different cultures, even without knowing every detail about each culture.

### 3.3. Factors Influencing Students’ Interpretations of Cultural Competence

This theme explores the important factors that may shape participants’ understandings of cultural competence, particularly within the context of cultural diversity among local and international nursing students in Australian tertiary education. Considering this, some factors were identified through nursing students’ reflections, particularly communication challenges and personal learning experiences.

A common communication issue raised by participants was the presence of intercultural communication barriers in both academic and workplace settings. Language incompetence, accent variations, and the use of local language or colloquialisms were barriers to greater intercultural communication. Participants described the Australian accent, and the use of slang was identified as an obstacle to effective communication, which could, in turn, affect their confidence and engagement with local peers.

In addition, participants noted that cultural differences could contribute to misunderstandings or discomfort when people from other cultural backgrounds failed to understand each other’s gestures, body language, words or facial expressions. Making assumptions or relying on stereotypes may lead to misunderstandings due to inadequate cultural knowledge and awareness. Similarly, participants reflected that interacting with people from diverse cultural backgrounds can be challenging, and sometimes stressful and difficult. These reflections suggest that intercultural communication is more than language proficiency; it requires sensitivity to verbal and nonverbal cues to respond and act appropriately.

Further, participants reported difficulties in interpreting patients’ perceptions of respect, which may vary depending on cultural norms. They perceived the complexity of intercultural interactions and differing cultural expectations within healthcare settings as significant communication challenges. For example, Participant 3 commented, “I worry about the best way to understand and support my patients with cultural backgrounds. It is not always easy to know how they perceive respect” (P3). As a result, students may sometimes experience stress and difficulty when conversing with people from diverse cultures.

Moreover, participants’ learning experience in nursing education, including clinical placements and clinical skills laboratory sessions, was an influential factor affecting their perceptions of cultural exposure and learning. Participants stated that they became more culturally sensitive and aware of cross‐cultural diversity when they had more opportunities to connect and collaborate with their peers, mentors, colleagues, and patients. For example, Participant 5 commented:

*The more experience I have working with other cultures and colleagues, the more I learn about cultural and cultural competence.* (P5)

Overall, participants’ responses suggest that an inclusive learning environment with regular multicultural engagement opportunities can facilitate positive experiences of intercultural interactions.

### 3.4. Learning Cultural Competence

This theme explores participants’ perceptions of how cultural competence is learnt and developed within nursing education. This theme integrated the participants’ descriptions of learning experiences, whether these occurred within their academic studies or through activities outside the university. Participants’ responses highlighted the need to review and reform cultural competence training in the current nursing curriculum, specifically cultural diversity training, to prepare them for clinical placements. They suggested a broader range of cultural training was required at institutional and clinical levels. For example, Participant 4 stated:

*I sometimes worry about not knowing the best way to care for patients from different cultures, especially when it comes to showing respect… I feel like the curriculum could be refined and more diversified to better prepare future nurses.* (P4).

This response suggests that being culturally competent can positively affect the quality of care, patients’ adherence to treatment plans, the effectiveness of therapeutic communication, and overall health outcomes.

Additionally, participants reported challenges with cross‐cultural learning during clinical laboratory sessions. They observed that students were unwilling to leave their social comfort zone. During the clinical laboratory sessions, students were more inclined to choose students with similar cultural backgrounds. For instance, Participant 4 commented, “walking into the laboratory room, I noticed that all the students were grouped based on their cultural background. I wished the facilitator had mixed us up to encourage interaction”. Consequently, students had limited exposure to cultural diversity, which may have reduced opportunities for cross‐cultural interaction within the simulated clinical learning environment.

Despite these challenges, participants generally valued the multicultural nature of the healthcare environment. Participants described how working with culturally diverse peers, mentors, and patients helped them to become more aware of individual uniqueness and reflect on their own values. For example, Participant 2 stated:

*I think it is good to have a combination of students who come from (diverse cultural backgrounds). Each person has their unique qualities and personality, which really helps me to learn more about people and how to work with them respectfully.* (P2)

Through these intercultural encounters, students become more culturally aware, enabling them to ethically align their personal and professional values within nursing practice, fostering respectful relationships and promoting an inclusive environment. In general, participants agreed that changes were needed in the learning environments to better support students’ cultural competence development. However, they also emphasised that meaningful change and deeper learning must begin from within each nurse.

## 4. Discussion

The aim of this study was to explore how undergraduate nursing students understand and define cultural competence, as well as the factors that may impact students’ interpretations of cultural competence. Within this context, the quantitative findings reveal participants perceived themselves as moderately culturally competent, consistent with recent studies [28]. However, the cultural sensitivity subscale had a notably low average score, indicating a possible gap among students in this specific area of cultural competency. Cultural sensitivity, a fundamental element of cultural competence, involves developing cross‐cultural communication skills [8]. The result suggests that linguistic barriers and negative emotions, such as lack of confidence and fear, significantly impede rapport and relationship‐building in classroom, laboratory, and clinical practice environments [11].

In addition, the study suggested that non‐Australian students had higher cultural sensitivity scores than Australian students. This may be attributed to students from minority backgrounds being more culturally sensitive and aware in multicultural environments, as they constantly self‐reflect on their cultural identity while living in their host countries [8]. Furthermore, male students had higher cultural competency scores than female students, which aligns with some studies [29] but contradicts others [30]. However, these findings must be interpreted cautiously due to the small sample size, particularly the limited number of male participants, which may have skewed the results. The quantitative results also show no clear association between age and cultural competence level, which is consistent with other studies [29, 30].

The qualitative findings provide deeper insights into the quantitative results, revealing that most participants had limited foundational skills in intercultural communication. This finding challenges the quantitative data and may arise from participants providing more in‐depth descriptions of their lived experiences during interviews or their self‐assessment on the questionnaire being above their actual competence. Participants reported encountering linguistic barriers and negative emotions, such as a lack of confidence and fear, which hindered rapport and patient safety. This finding is consistent with previous research, which found that nursing students expressed concerns about patients not fully understanding health‐related education and failing to establish rapport due to poor therapeutic communication [31, 32].

Higher cultural sensitivity scores among non‐Australian students were further explained by their strong motivation and determination to immerse themselves in a new culture. This group of students demonstrated a continuous interest in improving their intercultural communication skills, which is critical for developing cultural sensitivity [33]. Additionally, the qualitative results of this study further reveal that this group of students has a strong sense of motivation and determination to immerse themselves in a new culture when studying abroad. Consistent evidence indicates that personality traits such as moral conscience, motivation to understand local culture, and professional commitment are critical to the development of cultural sensitivity [33]. Likewise, Garneau and Pepin [34] and Henderson et al. [7] reported similar findings, indicating that commitment initiates student nurses’ desire to become more knowledgeable and competent in delivering culturally diverse care.

Overall, the study suggests that students perceive cultural competence as a continuous process of growth and learning, essential to providing compassionate and culturally competent care. This aligns with previous studies that define cultural competence as healthcare providers’ ability to continuously engage with their patients’ cultural contexts [20]. Participants highlighted the need for broader cultural training in nursing curricula to address gaps between taught content and practical needs. Institutions should support international students’ integration and promote cross‐cultural activities to enhance cultural competence. However, there is limited consensus among researchers regarding the teaching strategies of cultural competence in nursing [32].

These findings also resonate with Campinha‐Bacote [20] model of cultural competence, which conceptualises competence as a process involving five interrelated constructs: cultural awareness, cultural knowledge, cultural skill, cultural encounters, and cultural desire. The quantitative results reflect high levels of awareness and knowledge, but lower scores in cultural sensitivity suggest gaps in skill and encounters, key areas for curriculum enhancement. The qualitative data further support this, with students expressing a desire to improve and acknowledging the importance of ongoing learning, which corresponds to the ‘cultural desire’ component of the model.

Similarly, the influence of educational and clinical experiences on students’ development of cultural competence echoes Leininger’s Culture Care Theory, which highlights the significance of cultural context and care diversity in health practices [35]. The findings suggest current curricula may not fully support this congruence, particularly in fostering intercultural communication skills. This highlights the need for curriculum design that integrates experiential learning, reflective practice, and diverse cultural encounters. Overall, the findings not only support the existing models but also extend them by illustrating how students’ cultural competence is shaped by peer dynamics, communication barriers, and institutional learning environments.

As such, evidence suggests that cross‐cultural immersion programs and web‐based teaching approaches are more effective than traditional contact teaching for developing cultural competence among nursing students [1, 30, 36]. Tee et al. [15] found that nursing students are receptive to working with people from different cultures. Regular intercultural interactions can help them to alleviate their anxieties and fears about feeling unprepared, while also enhancing their self‐awareness and confidence in providing person‐centered care [15]. This is similar to other studies, which found that participants preferred more intercultural encounters within healthcare institutions and clinical settings. As such, learning and teaching efforts should focus on immersing students in various migrant communities [1, 37] and encouraging intercultural encounters through extracurricular activities. This may improve cultural competency [38].

Additionally, previous studies have shown that an online cross‐cultural learning approach is an alternative and effective option compared to studying abroad [1]. According to a previous literature review, it provides a safe environment for students to mutually share their own cultural values and beliefs, fostering cultural sensitivity through communication [39]. Moreover, web‐based interaction with international peers may help students view nursing through an intercultural lens [1], thereby transforming their understanding of the nursing profession and enabling a far greater global perspective [40]. Therefore, students will be able to reflect on how they might ethically align their personal and professional values in nursing practice.

### 4.1. Limitations

A limitation of the study is its small sample size, with 25 students completing the questionnaire, and five participating in interviews. While this limits the generalisability and statistical power of the findings, the study was designed as an exploratory investigation using an explanatory sequential mixed‐methods approach. The qualitative phase aimed to provide in‐depth insights into students’ perceptions and learning experiences, rather than produce generalisable outcomes. Additionally, the sample had limited demographic diversity, particularly a small proportion of international students, which may restrict the applicability of findings to broader nursing student populations. For instance, the higher cultural competence scores among male students should be interpreted cautiously, as the small number of male participants may have introduced sampling bias. Future research with larger and more diverse cohorts is recommended to validate and expand upon these findings.

In addition, the low response rate in the survey may be due to the survey topic being less compelling, third‐year students’ busy schedules, or post‐COVID fatigue from the rapid shift to online learning [41]. Additionally, the lack of incentives for the lengthy survey may have contributed to low participation. These factors may indicate potential biases or a lack of engagement with the study topic. Within this context, future research should consider offering incentives and engaging potential participants to increase response rates.

Finally, while focusing on nursing students is relevant within this study, the findings may have limited transferability beyond this cohort. However, the insights gained may offer valuable implications to other tertiary institutions where cultural education is a curricular focus. Thus, participants’ perspectives may be transferable to student nurses with similar cultural and educational backgrounds situated in other geographical regions across the country. Future studies could expand the study to include second‐year students or those from other health‐related disciplines, such as paramedics, psychology, and allied health, to provide a more comprehensive understanding of cultural competence in health education. This broader inclusion may improve the transferability of the findings.

### 4.2. Future Implications

To date, there is limited research on intercultural learning interventions aimed at improving student nurses’ cultural competence. Given the constantly changing global demographics of nursing students in the Australian tertiary education sector, future research may benefit from conducting longitudinal studies with larger cohorts. These studies could focus on evaluating the long‐term impact of educational interventions on students’ cultural competency in nursing practice. Researchers may also explore other pedagogical strategies, including online international learning, immersive cultural experiences, and international collaborative partnerships in nursing education. These strategies may assist educators in higher education, specifically nursing programmes, in identifying effective teaching strategies to better equip nursing students to provide safe and competent care to patients with diverse cultural backgrounds after graduation. Similarly, a prospective study could be adopted to illustrate the longitudinal transition of cultural competence from student nurses to graduate nurses.

Specifically, in today’s global climate, the internationalisation of curriculum has become a leading trend and focus in Australian higher education [42]. Integrating cross‐cultural learning into web‐based teaching approaches is becoming a prevailing direction in nursing education. Additionally, previous research shows that virtual learning can optimise students’ intercultural experience [43] and deepen their understanding of issues related to culture, health and the health system from a global perspective [44]. However, there is insufficient research investigating the effectiveness of collaborative online international learning. Future studies may benefit from examining how this learning approach impacts student engagement, learning outcomes, and cultural competence development. Furthermore, understanding the long‐term benefits of collaborative online international learning on students’ cultural skill development and global health education would be beneficial. These insights may be valuable to educators as they develop future courses and nursing programmes.

In addition to these broader pedagogical directions, nursing curricula should incorporate intercultural exposure early in the programme, such as through orientation activities, cultural awareness workshops, and peer‐led intercultural dialogues. Structured mentorship programmes that pair domestic and international students so as to foster mutual learning and reduce social segregation. Scenario‐based training that simulates culturally diverse patient interactions should be embedded in clinical preparation to enhance students’ confidence and communication skills. Moreover, faculty development initiatives are essential for equipping educators with the tools to facilitate inclusive learning environments and model culturally competent behaviours. These actionable strategies can help bridge the gap between theoretical knowledge and practical application, ensuring nursing graduates are well‐prepared to deliver culturally safe and responsive care.

## 5. Conclusion

This research has identified that certain demographic factors, such as gender and cultural background, may influence students’ level of cultural competence and cultural sensitivity. However, given the small sample size, this finding needs to be interpreted cautiously and considered as exploratory rather than conclusive. These differences may be attributed to personal and social factors, including intercultural communication barriers and social segregation. Enhancing cultural competence among nursing students is the overarching theme for continuous professional development. It is worth noting that cultural encounters under guidance and supervision can be an effective approach to learning cultural competence. Through collaborative online international learning, students can gain a broader understanding of cultural diversity, broaden their vision to a global perspective, and reflect on how they can ethically integrate their personal and professional values into their nursing practice.

## Funding

Open access publishing facilitated by Federation University Australia, as part of the Wiley ‐ Federation University Australia agreement via the Council of Australasian University Librarians.

## Conflicts of Interest

The authors declare no conflicts of interest.

## Supporting Information

Supporting information 1: Individual interview schedule.

## Supporting information


**Supporting Information** Additional supporting information can be found online in the Supporting Information section.

## Data Availability

The data that support the findings of this study are available from the corresponding author upon reasonable request.
