# Supplementary material for: Exploring the Meaning of Cultural Competence Among Undergraduate Nursing Students
Source: Nurs Res Pract. 2026 Feb 15;2026:3963409. doi: 10.1155/nrp/3963409 (PMC12907568; doi:10.1155/nrp/3963409)
Supplement: Supplementary file 1 — Supporting Information Additional supporting information can be found online in the Supporting Information section. [file NRP-2026-3963409-s001.docx]

**Supplementary file 1: Individual Interview Schedule**

1. What is the first thing that comes to your mind when talking about culture?

2. Please describe in your own words what ‘cultural competence‘ means to you.

3. What is your experience with cultural differences and learning during your nursing

education?

4. What differences, if any, did you experience between your culture and the culture you

are living in this nursing education context?

5. When is a time you remember one episode (or more) that best reflects these

differences?

6. How does this experience impact on your meaning of cultural competence?

7. What are the other factors that may influence your definition of cultural competence?

8. Can you tell use more about how did (this or these) factor(s) influence your definition

of cultural competence?

9. Is there anything else that you would like to add that we may have not covered?

Prompting questions were used to delve deeper into the experiences throughout the interview:

- Can you tell us more about your reactions to…?
- How did (this or that) happen?
- Can you tell me how it changed your meaning of cultural competence?
